# Assessing the impact of workaholism and work engagement on medical university employee stress and satisfaction levels

**DOI:** 10.7717/peerj.12565

**Published:** 2022-02-04

**Authors:** Mahin Sarfaraz, Shaur Sarfaraz, Afsheen Maqsood, Naseer Ahmed, Fahim Vohra, Tariq Abduljabbar, Adel S. Abduljabbar

**Affiliations:** 1 Institute of Health Management, Dow University of Health Sciences, Karachi, Sindh, Pakistan; 2 Institute of Medical Education, Jinnah Sindh Medical University, Karachi, Sindh, Pakistan; 3 Bahria University Dental College, Karachi, Pakistan; 4 Department of Prosthodontics, Altamash Institute of Dental Medicine, Karachi, Pakistan; 5 Department of Prosthetic Dental Science, College of Dentistry, King Saud University, Riyadh, Saudi Arabia; 6 Department of Psychology, College of Education, King Saud University, Riyadh, Saudi Arabia

**Keywords:** Job performance, Medical employees, Workaholism, Work engagement, Wellbeing

## Abstract

**Background:**

Workaholism (WH) is related with high mental trouble and physical objections, low employment and family fulfillment, and low occupation execution; however, work commitment is related with job and life satisfaction. This study aimed to assess the impact of WH and work engagement (WE) on medical university employee job stress and satisfaction.

**Methods:**

This descriptive analytical study was conducted on workers employed in medical universities using validated questionnaires for data collection. The sample size was 330, from which 305 responded with completed questionnaire. The employees were the direct workers of different accessible medical universities in Karachi, Pakistan. The employee enrolment and distribution of the questionnaire was performed using non-probability convenient sampling. The Brief Job Questionnaire (BJQ), Dutch Work Addiction Scale (DUWAS) and Utrecht Work Engagement Scale (UWES) were used to assess the impact of job stress on job satisfaction and WE. The data was analyzed by applying descriptive statistics, Spearman correlation and regression analysis. A *p*-value of ≤ 0.05 was taken as significant.

**Results:**

The study consisted of 117 (38.4%) male and 188 (61.6%) female participants; the mean age of participating employee was 28.50 ± 6.192. The mean score for WE was 3.78 ± 0.467, that of WH was 3.42 ± 0.559, for JS was 4.29 ± 0.400, whereas JSF was 3.10 ± 0.591. A positive correlation was observed between job stress, WH and WE. WH had a strong correlation with job stress and weak correlation with job satisfaction and performance. A significant difference was observed between WE and WH among males and females.

**Conclusions:**

The study presented with a significant effect of job satisfaction with WH and WE with job stress. Hence, indicating the importance of social skills and WE with fellow employees to increase the work productivity and performance. However, in case of over work and WH, an increase of job stress is inevitable.

## Introduction

The understanding of work and its temperament has been extensively studied recently, due to alterations in work dynamics, business and vulnerability of work conditions (Taris, Schaufeli & Shimazu, 2010). Work engagement (WE) and workaholism (WH) are interconnected, yet perceived as different concepts (Bakker, Demerouti & Sanz-Vergel, 2014). Bakker, Demerouti & Sanz-Vergel (2014) characterized workaholics as individuals who work extra hours without any particular request. These compulsive workers give special preference towards work irrespective of time, working hours and labor category (Nishi et al., 2016). Furthermore, Guindon & Cappeliez (2010) described three types of work extremists; the Enthusiastic–subordinate, stickler and accomplishers in an organization. Irrespective of the high performance rate, these employees display signs and symptoms of overwork.

The high predominance of over-work has prompted an increase in concern of public health regarding negative influence on health. The extended working hours cause sleep deprivation, decrease in neurocognitive and physiological working, weak task execution, and an expanded danger of health deterioration (Cappeliez & Robitaille, 2010). From an extensive point of view, current financial retreat, authoritative retrenchment and developing occupation frailty may continuously urge representatives to contribute an expanding measure and exertion into their work to improve employment (Cappeliez & Robitaille, 2010). Studies further identified that employees with burnout often suffer a range of physical health problems (Bakker, Demerouti & Sanz-Vergel, 2014; Park, 2020). Due to sleep deprivation and overload of work, increases the risk of stroke along with mental health and greater chance for depression, anxiety and suicide.

WE are when a person discovers joy in work in spite of strenuous employment requests and stressors (Ungar & Theron, 2020; Gostin, Constantin & Meier, 2020). Nishi et al. (2016) demonstrated that WE is an indicator of wellbeing, higher life fulfillment, and predominant occupation execution, with low depressive disorders. Henceforth, WE includes performing task without stressing and interchanging ideas and plan for better performance and outcomes (Gostin, Constantin & Meier, 2020; Atroszko & Atroszko, 2020). Moreover, WE promotes the state of being comfortable, healthy, or happy and a stress free life (Sharma, 2017). Thus, an employee with maximum social skills easily adheres to the work environment and fulfills all their responsibilities (Dospinescu & Dospinescu, 2020; Otsuka et al., 2020). Similarly, it was observed that workaholics are less happy with their employment despite of being energetic. Pocnet et al. (2014) revealed that satisfaction towards a job could be estimated in view of psychological wellbeing, emotional feelings and social interaction of the employee at work.

To understand the relation of work stress with health and well being of an individual, it is important to understand the concept of work stress in order to design a proper intervention for the workplace. The impact of work stress is not only related to the physical health but also influences mental status that elicit psychological distress (Pocnet et al., 2014). Recently, researchers have predicted that over work and work-stress has led to increase in the global and national recession, job insecurity and work intensity that causes excessive workloads and more interpersonal conflicts among the employees indirectly (Orgambídez-Ramos, Borrego-Alés & Mendoza-Sierra, 2014). Furthermore, a brief comparison using theoretical models demonstrated that increasing stress is commonly associated with adverse life events and stressful environments, that intern compromises work life and psychological response, indirectly influencing work engagement (Coetzee & De Villiers, 2010; Schmitt, Den Hartog & Belschak, 2016).

Some authors have analyzed that positive work culture and good management is critical for instigating a positive attitude among the employees, which in return enhances their performance and supports their well being (Coetzee & De Villiers, 2010; Schmitt, Den Hartog & Belschak, 2016). Therefore, in light of the effects of stressful work environment on employee wellbeing in medical institutions, the current study was planned to assess an association between WE and WH and their effect on job satisfaction and job stressors. The null hypothesis was that WE and WH are not associated and do not influence job satisfaction and job stressors. The present study aimed to assess the impact of WH and WE on medical university employee job stress and satisfaction.

## Materials and Methods

### Ethical consideration

The study protocol was prepared in line with the standards of the Helsinki declaration (1964) and was approved by the institutional review board of Altamash Institute of Dental Medicine (AIDM/ERC/11/2019/02). Subsequent to the approval of the protocol, the enrolled participant’s written consent was obtained for their voluntary participation. Each employee was given the right to withdraw without consequences. The information collected was kept restricted to this research, with maintenance of confidentiality and anonymity of the research respondents and there voluntary participation.

### Study design, Settings and sampling technique

This was a cross-sectional study that was conducted over 8 months. The sample size of the study was 330, from which only 305 responded to the questionnaire. The participants were the direct employees of three medical universities which had both medical and dental schools in Karachi, namely Karachi Medical and Dental University, Jinnah Sindh Medical University, and DOW University. The employee enrolment and distribution of the questionnaire were performed based on non-probability convenient sampling technique.

### Questionnaire and variables

The study was conducted using modified questionnaires on workaholism, work engagement, job stress and satisfaction questionnaire (WHWEJSSQ), taking guide from three validated research tools, The Brief Job Questionnaire (BJSQ), Dutch Work Addiction Scale (DUWAS) (Dospinescu & Dospinescu, 2020) and Utrecht Work Engagement Scale (UWES) (Dospinescu & Dospinescu, 2020; Otsuka et al., 2020). The WHWEJSQ consisted of 76 closed ended questions. The items were divided into three sections. The work engagement section consisted of 11 questions regarding vigor, dedication and absorption to the work. Similarly WH section contained 13 questions consisting of statements with compulsiveness. The responses were collected on a five point Likert scale, 5 = Always, 4 = Frequently, 3 = Sometimes, 2 = Infrequently, 1 = Never. The Maximum score for work engagement was 55, out of which 1–18 denotes poorly engaged, 19–36 means moderately engaged and 37–55 suggests highly engaged. The Maximum score for workaholism was 65, where 1–21 denotes less workaholic, 22–42 means moderately workaholic and 43–65 suggests highly workaholic. Lastly, the job stress (JS) section consisted of 40 questions emphasizing the health conditions, which represents the wellbeing of an employee. The maximum score for JS was 200, where 1–66 means not stressed, 67–132 suggests moderately stressed, 132–200 was categorized as highly stressed.

The Job Satisfaction (JSF) section consisted of 12 questions. The maximum score for JSF was 44. The responses were recorded on four point Likert scale, 4 = Extremely, 3 = Very much, 3 = Somewhat, 1 = Not at all. A score of 1–15 was categorized as not satisfied, 16–30 somewhat satisfied and 31–44 very satisfied. The Cronbach’s alpha analysis was performed to check the internal consistency of items in three questionnaires adopted for this study and a strong Cronbach’s Alpha value of 0.81, was recorded.

### Data collection procedure

The survey questionnaires were distributed at different medical and dental institutes. The employees were asked to complete the questionnaire on paper. In circumstances with employees having busy schedules, a drop off and pick was deployed to collect information from respondents. A total of 330 questionnaires were distributed to participants, however 305 completed questionnaires were received and included in the study.

### Statistical analysis

Data were analyzed using statistical program for social sciences (SPSS, Version 25 IBM, NY, USA). Descriptive statistics were carried out to calculate percentage (%), frequency (f), arithmetical average, range and standard deviation of qualitative variables (gender, designation and items of questionnaires) and quantitative variables (Age, Mean item scores and categories of the questionnaire). Spearman correlation coefficient (r), Independent t test, and multiple regression analysis were used to detect association and effect for each dependent and independent variable. A *p*-value ≤ 0.05 was taken as statistically significant.

## Results

The present study demonstrated a positive correlation between job stress, WH and WE. A significant difference was observed among the study groups with respect to gender. This analytical study evaluated a total of 305 employee and presented a successful response rate of 92.42%.

Table 1 showed the description of each participating employee in relation to WE WH, JS, and JSF along with its gender disparity. The study consisted of 117 (38.4%) male and 188(61.6%) female employee; the mean age of employee was 28.50 ± 6.192. The mean score for WE were 3.85 ± 1.06 (total score; 42.36 ± 11.702), while that of WH was 3.42 ± 1.19 (total score; 44.5 ± 15.52) and JS was 3.11 ± 1.18 (total score; 124.65 ± 47.291). The mean score of JSF was 3.10 ± 0.591 (total score; 37.2 ± 7.141). The detailed distribution of the WE, WH, JS and JSF are presented in Appendix A.

**Table 1:** Distribution of WE, WH, JS, and JSF attributes amongst the employees (mixed group), male (*n* = 117), female (*n* = 188).

WHWEJSSQ whole scales score	Mean	SD	Gender disparity (Mean and SD)		*p* value
			Male	Female	
WORK ENGAGEMENT (WE) *Average (total score)	3.78 (42.36)	0.467 (11.702)	3.88 ± 0.455 (43.53 ± 9.441)	3.72 ± 0.465 (41.62 ± 12.186)	0.004^β^
WORKAHOLISM (WH) **Average (total score)	3.42 (44.5)	0.559 (15.52)	3.52 ± 0.530 (45.7 ± 15.486)	3.36 ± 0.570 (43.72 ± 15.471)	0.020^β^
JOB STRESS (JS) ***Average (total score)	4.29 (124.65)	0.400 (47.291)	4.29 ± 0.373 (124.42 ± 46.725)	4.29 ± 0.417 (124.78 ± 47.381)	0.901
JOB SATISFACTION (JSF) ****Average (total score)	3.10 (37.2)	0.591 (7.141)	3.09 ± 0.55 (37.08 ± 6.689)	3.10 ± 0.58 (37.25 ± 7.348)	0.072

**Notes:**

WE, Work engagement; WH, Workaholism; JS, Job Stress, JSF, Job satisfaction.

*Highly engaged (WE) score, **Highly workaholic (WH) score, ***Moderately stressed (JS) score, ****very satisfied (JSF) score; Independent t-test.

β *p* < 0.05.

The mean of WE among male and female employees was 3.88 ± 0.455 (total score; 43.53 ± 9.441) and 3.72 ± 0.465 (total score; 41.62 ± 12.186) respectively. WE was significantly different among male and female employees (independent-t test; *p* = 0.004). The WH mean score in males was 3.52 ± 0.530 (total score; 45.7 ± 15.486), while 3.36 ± 0.570 (total score: 43.72 ± 15.471) among females. A significant difference was seen between both gender (independent t-test; *p* = 0. 0.020) for WH. Moreover, the mean score of JS in male employee was 4.29 ± 0.373 (total score; 124.42 ± 46.725), whereas in females it was 4.29 ± 0.417 (total score; 124.78 ± 47.381), however no significant difference was found (independent-t test; *p* = 0.0901). The mean score of JSF amongst male employee was 3.09 ± 0.55 (total score; 37.08 ± 6.689), and in females the score was 3.10 ± 0.58 (total score; 37.25 ± 7.348) however no significant difference was observed (independent t-test; *p* = 0.072).

In terms of job satisfaction, the analysis of JSF scale items revealed that, each participant showed poor communication skills with their colleagues as the level of superiority increased. The outcome showed low trust, discouraging workplace attitude, low workplace engagement and increased level of dissatisfaction among the employee. Hence, these employees reported less satisfaction with their job compared to their family life.

A weak correlation was observed between WE and WH (Spearman correlation; rho = 0.254, *p* = 0.001). Similarly, a weak correlation was also found between WE and JS (Spearman correlation; rho = 0.159, *p* = 0.005). Whereas a strong correlation was found between WE and JSF amongst the employees (Spearman correlation; rho = 0.717, *p* = 0.003). A weak correlation was found between WH and JSF (Spearman correlation; rho = 0.148, *p* = 0.010). And a strong correlation was seen between WH and JS (Spearman correlation; rho = 0.893, *p* = 0.001) as presented in Table 2.

**Table 2:** Correlation between Work engagement (WE), Workaholism (WH) with job stress and job satisfaction.

Variables		Correlation	*p*-value
1	Work engagement	0.254	0.000
	Workaholism		
2	Work engagement	0.159	0.005
	Job stress		
3	Work engagement	0.717	0.003
	Job satisfaction		
4	Workaholism	0.893	0.001
	Job stress		
5	Workaholism	0.148	0.010
	Job satisfaction		

Table 3 presents regression analysis of job satisfaction and job stress with respect to work engagement and work stress. The outcome of the study showed that a positive correlation between job satisfaction and WE; and job stress and WH existed. The regression analysis for job satisfaction to WE showed a R-squared value of 0.21 and adjusted R square of 0.049, similar to WH; however, under influence of external variables only WE presented a significant difference (*p* = 0.013). WE beta value was statistically significant (B = 0.186, *p* = 0.01) which indicates that an increase in WE score will ultimately increase job satisfaction by 0.186. Whereas, on average the job satisfaction was, B_0_ = 2.165 in this study. Moreover, for job stress the R-square value was 0.227 and adjusted R square was 0.048 with respect to the WH and WE respectively. Under the influence of the external variables, only job stress and WH showed a positive correlation in contrast to WE. The WH beta value was statistically significant (B = 0.118, *p* = 0.016), indicating that an increase in WH scores will ultimately increase job stress. While on average the job stress was, B_0_ = 3.50 amongst participants.

**Table 3:** The regression analysis of independent variables Job stress and job satisfaction levels, age and gender with work engagement and workaholism amongst the participants.

Variables	Work engagement (WE)					Workaholism (WH)				
**Job satisfaction**	**R**	**AR** ^**2**^	**B**_**0**_	**B**	***p*-value**	**R**	**R** ^**2**^	**B**_**0**_	**B**	***p*-value**
	0.215	0.049	2.165	0.186	0.013	0.219	0.049	2.16	0.111	0.062
**Job stress**	0.227	0.048	3.501	0.106	0.069	0.225	0.048	3.50	0.118	0.016
**Gender**	0.165	0.024	0.159	0.165	0.004	0.133	0.014	0.151	0.133	0.020
**Age**	0.046	−0.001	0.003	0.046	0.424	0.027	−0.003	−0.002	−0.027	0.639

**Note:**

R-Squared (R): Regression model predicted and observed values of WH, WE with Job satisfaction, stress, age, and gender. Adjusted R-Squared (AR^2^): predictability of variance in dependent and independent variables, B, Beta = denotes the correlation between dependent and independent variables, B_0_ = unstandardized coefficient *i.e*., average estimation of stress, satisfaction, age, and sex with WE, WH. *p*-value < 0.05 is considered significant.

In the regression analysis of gender and WE, the constant for R-Squared (R) was 0.165 and adjusted R-Squared (AR^2^) was 0.024. The correlation of gender with WE was significant (B = 0.165, *p* = 0.004) and the average of gender with WE relation was 0.159 (Bo). Similarly, for gender and WH, the constant for R-Squared (R) was 0.133 and adjusted R-Squared (AR^2^) was 0.014. A significant correlation was found between gender and WH (B = 0.133, *p* = 0.020); and average gender with WH regression was 0.151 (Bo). The significant values indicate that the level of WH and WE were more in males as compared to females.

Furthermore, in analysis of age and WE the constant for R-Squared (R) was 0.046 and adjusted R-Squared (AR^2^) was −0.001. The correlation of age with WE was insignificant (B = 0.046, *p* = 0.424) and average age with WE relation was 0.003 (Bo). For gender and WH the constant for R-Squared (R) was 0.027 and adjusted R-Squared (AR2) was −0.003. The correlation of age with WH were, −0.027 (B) (*p* = 0.639) and average age with WH relation was −0.002 (Bo).

## Discussion

The present study assessed the impact of WH and WE on university employee job stress and job satisfaction (wellbeing) levels. Job satisfaction is considered as a positive factor in instilling an optimistic attitude and improves WE among the employees; however, few factors such as job stress, lack of peer relationships, and dissatisfaction with job, negatively influences the WE and productivity of the employees. The study outcomes demonstrated that workaholism and work engagement were strongly associated with one another and their relationship with wellbeing was distinct; nevertheless, extreme behavior displayed a negative effect on health whereas an active work environment promoted good health amongst employees. Hence, the null hypothesis was rejected.

The present study used a combination of three questionnaires and scales to assess the impact of workload and job satisfaction on work engagement and health. Each questionnaire has its own uniqueness and covers a specific area of interest. BJSQ is a simple questionnaire designed to specifically target highly stressed workers. The BJSQ consists of 57 items aimed to assess two specifications; job stressors (psychological job demands, job control), psychological and physical stress reactions and buffering factors, such as social support at work. On the other hand, Dutch Work Addiction Scale (DUWAS) is used to measure the level of work addiction among the employee. Whereas UWES is a questionnaire to measure the three constituting aspects of WE: vigor, dedication, and absorption. Considering the present study aim, all three questionnaire outcomes were combined to interrelate the impact of the job stressor and WH on health and WE.

Stress is a trigger factor, which produces a burnout effect on professional performance (Orgambídez-Ramos, Borrego-Alés & Mendoza-Sierra, 2014). Excessive stress buildup has shown to increase 3 times the risk of workplace absentees, sickness, increase turnover rates, impairs strategic thinking and dulls creative abilities. Studies have shown that the enhanced employee engagement is an excellent predictor of work performance, organizational success, financial performance, and client satisfaction (Coetzee & De Villiers, 2010; Schmitt, Den Hartog & Belschak, 2016). However, Dospinescu & Dospinescu (2020) termed this as an occupational hazard where increase work pressure engagement impairs physical health, psychological well-being, and work performance. Hence, job stress and pressure acts as a mediator between demands and work related outcomes. In the present study, WH was related to poor health, life unhappiness, poor employment performance, as WH beta values were significant (β = 0.118, *p* = 0.01). Suggesting that increase in WH scores will ultimately increase job stress (poor health), whereas job and life satisfaction showed low relationship, as the beta value was, 0.111. This implies that WH and WE are two distinct attributes, and judicious use of both parameters is warranted at the workplace.

Furthermore, the study suggests that work overload is related to bad health, while WE enhances the well being or prosperity of an employee (Mazerolle & Eason, 2017; Lorente, Tordera & Peiró, 2018). The present study identified a distinct relationship between job satisfaction, health, wellbeing and WE. Suggesting that WE showed association with quality of life, job satisfaction and low values with ill health (β = 0.10). Analyzing the outcomes, the strong relationship of WE with life fulfillment (β = 0.186) underlines the motivational part of WE. In a workplace environment, especially office work, continuous sitting with increased screen time for work, raises the risk of health-related problems. Studies have demonstrated that high personal energy, mental resilience, and persistence in work are related to the physical, psychological, social, or organizational resources of each individual; however, excessive stress and negativity in the environment raises chances of sickness absenteeism, burnout and high job demand (Nasurdin, Ling & Khan, 2018; Innanen, Tolvanen & Salmela-Aro, 2014). In addition, interventional studies have shown positivity in work life by reducing sitting behavior and promoting standing and light movement (Lorente, Tordera & Peiró, 2018; Stoeber & Damian, 2016). Therefore, greater stress is placed on WE to promote an employee’s wellbeing and reduce mental stress and physical protestations.

To avoid WH, work delegation and setting a priority list of work targets need to be adopted and enforced by the employer to favor work engagement (Schou Andreassen, Ursin & Eriksen, 2007). It has been discovered that activity assets (*e.g*., self-governance, execution criticism, social help, supervisory instructing) and individual assets (*e.g*., self-adequacy, flexibility, confidence, hopefulness) are precursors of WE (Lo Presti, Kertechian & Landolfi, 2020). Along with this expanding work, assets may also positively affect WE. This can be accomplished by participative administration, expanding social help, getting positive criticism from authorities, and workgroup building (Schou Andreassen, Ursin & Eriksen, 2007). On the other hand, some studies have shown gender discrimination to be associated with WE. Men were observed to have longer working hours and senior job positions, which increases the level of stress among the employees (Sharma, 2017; Otsuka et al., 2020). Moreover, It was discovered that men with lower job grades had less sitting time, which increases their interaction with other employees (Sharma, 2017). Hence, the difference in gender influences the personality of individuals and level of confidence to manage job stress, which may interfere with work engagement.

Work addiction and commitment are both conceivable approaches to enhance representatives’ health (Schaufeli, Taris & Van Rhenen, 2008; Sheridan & Slocum, 1975). It was sought that work culture plays a pivotal role in improving business outcomes and propel growth. Strong work culture is supposed to enhance WE as it motivates, supports and encourages the employees to push towards higher goals (Bond & Bunce, 2003). If a company has developed a stronger work culture, then the system is well developed and organized, which instigates better understanding within the employees (Schaufeli, Taris & Van Rhenen, 2008; Rose, Kumar & Pak, 2009). These employees are more satisfied and actively engage with other members. On the other hand, workplaces practicing authoritative culture, believe that employees with greater working hours (workaholics) are the best people compared to the ones working with a sense of work-life balance (Wofford, 1971; Shore & Martin, 1989). Hence to improve health conditions and to combat work addiction, management should train employees to cope better with job stress and boost their ability to understand the complexity of work and acknowledge new tasks before finishing previous ones.

Within clinical perspectives, the study demonstrated a significant impact of overload work and job stress to negatively impact health and work engagement. However, some limitations were associated with the study, including limited participants. In addition, the study outcomes cannot be generalized as the work culture varies geographically, demographically and individual disciplines. In addition, genders were not equally distributed, which influences the outcome of the study providing a bias conclusion for gender influence on work engagement and job satisfaction. Therefore further multi center randomized controlled trials with a wider geographic, demographic and professional cohort and equal gender distribution, investigating the association of work overload and job stress on work engagement and performance are recommended.

## Conclusions

The study presented a significant effect of job satisfaction with work engagement and workaholism with job stress. A positive correlation was observed between job stress, WH and WE. WH had a strong correlation with job stress and weak correlation with job satisfaction and performance. A significant difference was observed between WE and WH among males and females. Hence, indicating the importance of social skills and work engagement with fellow employees to increase work productivity and performance. However, in the case of overwork and workaholism, an increase in job stress is inevitable.

## Supplemental Information

10.7717/peerj.12565/supp-1 Supplemental Information 1 Raw data.

10.7717/peerj.12565/supp-2 Supplemental Information 2 Detailed distribution of WE, WH, JS, and JSF attributes amongst the employees (mixed group), male (*n* = 117), female (*n* = 188).

## Additional Information and Declarations

### Data Availability

The following information was supplied regarding data availability: The raw data is available as a Supplemental File.
