# Supplementary material for: Assessing the impact of workaholism and work engagement on medical university employee stress and satisfaction levels
Source: PeerJ. 2022 Feb 4;10:e12565. doi: 10.7717/peerj.12565 (PMC8820209; doi:10.7717/peerj.12565)
Supplement: Supplemental Information 2 [file peerj-10-12565-s002.docx]

Appendix A: Detailed distribution of WE, WH, JS, and JSF attributes amongst the employees (mixed group), male (n=117), female (n=188).

| **Q** | | **WORK ENGAGEMENT (WE)** | **Mean** | **SD** | | **Gender disparity (Mean and SD)** | | **P value** |
| --- | --- | --- | --- | --- | --- | --- | --- | --- |
|  |  |  |  |  |  | **Male** | **Female** |  |
| 1. | | At my job, I feel strong and full of energy | 3.80 | 0.914 | | 3.73±0.738 | 3.85±1.008 | 0.248 |
| 2. | | I find my work meaningful and purposeful | 4.10 | 0.988 | | 4.09±0.799 | 4.11±1.091 | 0.879 |
| 3. | | Time flies when I am working | 3.92 | 1.140 | | 4.19±0.601 | 3.75±1.347 | 0.001^α^ |
| 4. | | When I am working, I forget everything else around me | 3.44 | 1.068 | | 3.67±0.938 | 3.29±1.121 | 0.003^β^ |
| 5. | | When I get up in the morning, I feel like going to work | 3.68 | 1.202 | | 3.91±1.071 | 3.53±1.256 | 0.006 ^β^ |
| 6. | | I feel happy when I am working intensely | 4.28 | 0.857 | | 4.32±0.829 | 4.25±0.875 | 0.460 |
| 7. | | I am proud on the work that I do | 4.27 | 0.952 | | 4.33±1.026 | 4.22±0.903 | 0.328 |
| 8. | | I can continue working for very long periods at a time | 3.88 | 1.049 | | 3.73±1.119 | 3.97±0.994 | 0.045 ^β^ |
| 9. | | To me, my job is challenging | 3.73 | 1.051 | | 3.68±0.999 | 3.77±1.083 | 0.438 |
| 10. | | It is difficult to detach myself from my job | 3.52 | 1.272 | | 3.70±1.227 | 3.41±1.291 | 0.052 ^β^ |
| 11. | | At my work I always stay positive, even when things do not go well | 3.74 | 1.209 | | 4.18±1.064 | 3.47±1.217 | 0.001 ^α^ |
|  | | *WE Average (total score) | 3.85 (42.36) | 1.062 (11.702) | | 3.95±0.946 | 3.78±1.107 | 0.0631 |
| **WORKAHOLISM (WH)** | | | | | | | | |
| 1. | | I often wish I were not so committed to my work | 2.64 | 1.249 | | 2.84±1.306 | 2.52±1.199 | 0.029 ^β^ |
| 2. | | I seem to be in a hurry and racing against the clock | 3.08 | 1.284 | | 3.27±1.375 | 2.96±1.212 | 0.040 ^β^ |
| 3. | | I find myself continuing work even though my co-workers quit | 3.59 | 1.079 | | 3.65±1.162 | 3.55±1.025 | 0.449 |
| 4. | | I work hard even I do not enjoy what I am doing | 3.53 | 1.365 | | 3.64±1.316 | 3.46±1.393 | 0.254 |
| 5. | | I think about work even when I want to get away from it for a while | 3.28 | 1.197 | | 3.27±1.134 | 3.29±1.238 | 0.923 |
| 6. | | I take too much work on me even if it becomes hectic | 3.37 | 1.160 | | 3.51±1.179 | 3.28±1.142 | 0.091 |
| 7. | | I seem to have a feeling that it is something I have to do whether I want to or not | 3.49 | 1.187 | | 3.59±1.131 | 3.42±1.219 | 0.226 |
| 8. | | I often feel that there is something inside me that drives me to work hard | 3.99 | 1.070 | | 3.85±1.127 | 4.07±1.026 | 0.070 |
| 9. | | I spend more time working than socializing with friends, on hobbies, or on leisure activities | 3.91 | 1.044 | | 4.06±0.967 | 3.81±1.081 | 0.045 ^β^ |
| 10. | | I feel guilty when I am not working on something | 3.77 | .950 | | 3.81±0.946 | 3.74±0.954 | 0.517 |
| 11. | | I feel obliged to work hard, even when it is not enjoyable | 3.76 | 1.047 | | 3.82±1.005 | 3.72±1.074 | 0.432 |
| 12 | | I find myself doing two or three things at one time such as eating lunch and writing a memo, while talking on the phone | 2.96 | 1.436 | | 3.11±1.437 | 2.86±1.430 | 0.140 |
| 13 | | It is hard for me to relax when I am not working | 3.13 | 1.452 | | 3.28±1.401 | 3.04±1.478 | 0.152 |
| **WH Average (total score) | | | 3.42 (44.5) | 1.196 (15.52) | | 3.51±1.187 | 3.363±1.189 | 0.0837 |
| **JOB STRESS (JS)** | | | | | | | | |
| 1 | | I have an extremely large amount of work to do | 3.37 | 1.047 | 3.57±0.994 | | 3.24±1.062 | 0.008 ^β^ |
| 2 | | I cannot complete work in the required time | 2.69 | 1.186 | 3.08±1.212 | | 2.44±1.105 | 0.001^α^ |
| 3 | | I have to work as hard as I can | 3.92 | 1.040 | 4.00±0.938 | | 3.87±1.099 | 0.278 |
| 4 | | I have to pay very careful attention | 4.20 | .889 | 4.03±0.850 | | 4.30±0.900 | 0.012 ^β^ |
| 5 | | My job is difficult in that it requires a high level of knowledge and technical skill | 4.08 | 1.087 | 4.01±1.110 | | 4.12±1.073 | 0.398 |
| 6 | | I need to be constantly thinking about work throughout the working day | 3.38 | 1.184 | 2.93±1.143 | | 3.66±1.124 | 0.001 ^α^ |
| 7 | | My job requires a lot of physical work | 3.69 | 1.076 | 3.38±1.015 | | 3.88±1.070 | 0.001 ^α^ |
| 8 | | I can work at my own pace | 4.00 | 0.997 | 4.10±1.163 | | 3.94±0.875 | 0.157 |
| 9 | | I can choose how and in what order to do my work | 3.83 | 1.112 | 3.82±1.291 | | 3.83±0.988 | 0.944 |
| 10 | | I can reflect my opinions on workplace policy | 3.20 | 1.109 | 3.00±1.182 | | 3.32±1.047 | 0.014 ^β^ |
| 11 | | My knowledge and skills are rarely used at work | 2.67 | 1.423 | 2.57±1.561 | | 2.73±1.331 | 0.352 |
| 12 | | There are differences of opinion within my department | 3.50 | 1.064 | 3.57±1.037 | | 3.45±1.081 | 0.337 |
| 13 | | My department does not get along well with other departments | 2.57 | 1.271 | 2.81±1.370 | | 2.41±1.183 | 0.008 ^β^ |
| 14 | | The atmosphere in my workplace is friendly | 4.11 | .920 | 4.13±0.952 | | 4.10±0.902 | 0.765 |
| 15 | | My working environment is poor (e.g., noise, lighting, temperature, ventilation) | 3.30 | 1.305 | 3.21±1.405 | | 3.36±1.239 | 0.326 |
| 16 | | This job suits me well | 4.14 | 0.953 | 3.89±1.024 | | 4.29±0.874 | 0.001^α^ |
| 17 | | My job is worth doing | 4.28 | 1.006 | 3.99±1.193 | | 4.46±0.823 | 0.001^α^ |
| 18 | | I have been very active | 3.67 | 1.213 | 3.87±0.961 | | 3.54±1.334 | 0.021^β^ |
| 19 | | I have been full of energy | 3.67 | 1.117 | 3.86±0.946 | | 3.55±1.198 | 0.016^β^ |
| 20 | | I have been lively | 3.69 | 1.185 | 3.76±0.887 | | 3.65±1.338 | 0.424 |
| 21 | | I have felt angry | 3.31 | 1.138 | 3.23±1.109 | | 3.37±1.155 | 0.310 |
| 22 | | I have been inwardly annoyed | 3.25 | 1.158 | 3.23±1.192 | | 3.27±1.139 | 0.797 |
| 23 | | I have felt irritable | 3.17 | 1.227 | 3.09±1.229 | | 3.23±1.226 | 0.322 |
| 24 | | I have felt extremely tired | 3.05 | 1.288 | 3.05±1.370 | | 3.05±1.238 | 0.982 |
| 25 | | I have felt worried or insecure | 2.71 | 1.276 | 2.81±1.238 | | 2.64±1.298 | 0.263 |
| 26 | | I have felt restless | 2.83 | 1.253 | 2.73±1.164 | | 2.89±1.304 | 0.258 |
| 27 | | I have been depressed | 2.69 | 1.122 | 2.56±1.070 | | 2.78±1.148 | 0.095 |
| 28 | | I have been unable to concentrate | 2.54 | 1.181 | 2.55±1.214 | | 2.54±1.163 | 0.944 |
| 29 | | I have been unable to handle work | 2.33 | 1.164 | 2.27±0.997 | | 2.37±1.257 | 0.496 |
| 30 | | I have felt sad | 2.90 | 1.243 | 2.86±1.299 | | 2.92±1.210 | 0.698 |
| 31 | | I have experienced joint pains | 2.50 | 1.403 | 2.46±1.424 | | 2.53±1.393 | 0.694 |
| 32 | | I have experienced headaches | 2.71 | 1.314 | 2.79±1.374 | | 2.65±1.276 | 0.364 |
| 33 | | I have had a stiff neck and / or shoulders | 2.50 | 1.306 | 2.41±1.092 | | 2.56±1.422 | 0.336 |
| 34 | | I have had lower back pain | 2.48 | 1.262 | 2.56±1.228 | | 2.44±1.284 | 0.423 |
| 35 | | I have had eyestrain | 2.45 | 1.312 | 2.36±1.323 | | 2.51±1.306 | 0.344 |
| 36 | | I have experienced heart palpitations or shortness of breath | 1.83 | 1.158 | 1.91±1.196 | | 1.78±1.134 | 0.344 |
| 37 | | I have experienced stomach and / or intestine problems | 2.20 | 1.296 | 2.30±1.205 | | 2.14±1.349 | 0.293 |
| 38 | | I have lost my appetite | 2.23 | 1.407 | 2.43±1.392 | | 2.11±1.406 | 0.053^β^ |
| 39 | | I have experienced diarrhea and / or constipation | 2.33 | 1.274 | 2.50±1.149 | | 2.22±1.338 | 0.069 |
| 40 | | I have not been able to sleep well | 2.68 | 1.326 | 2.74±1.233 | | 2.64±1.382 | 0.523 |
| ***JSQ Average (total score) | | | 3.11 (124.65) | 1.185  (47.291) | 3.08±1.142 | | 3.10±1.74 | 0.0951 |
| **JOB SATISFACTION (JSF)** | | | | | | | | |
| How freely can you talk with the following people? | | |  | | | | | |
| 1 | Superiors | | 2.98 | 0.598 | 2.99±0.549 | | 2.97±0.628 | 0.741 |
| 2 | Co-workers | | 3.59 | 0.512 | 3.57±0.497 | | 3.60±0.523 | 0.638 |
| 3 | Spouse, family, friends, etc. | | 3.40 | 0.657 | 3.32±0.667 | | 3.45±0.648 | 0.100 |
|  | How reliable are the following people when you are troubled? | |  | | | | | |
| 4 | Superiors | | 3.12 | 0.866 | 3.16±0.707 | | 3.09±0.952 | 0.481 |
| 5 | Co-workers | | 3.22 | 0.832 | 3.26±0.724 | | 3.19±0.893 | 0.454 |
| 6 | Spouse, family, friends, etc. | | 3.62 | 0.580 | 3.61±0.525 | | 3.62±0.613 | 0.821 |
| How well will the following people listen to you when you ask for advice on personal matters? | | |  | | | | | |
| 8 | Superiors | | 3.08 | 0.889 | 3.00±0.809 | | 3.13±0.933 | 0.223 |
| 9 | Co-workers | | 3.31 | 0.826 | 3.35±0.746 | | 3.29±0.873 | 0.517 |
| 10 | Spouse, family, friends, etc. | | 3.73 | 0.493 | 3.76±0.503 | | 3.71±0.488 | 0.410 |
| 11 | I am satisfied with my job | | 3.25 | 0.541 | 3.25±0.490 | | 3.25±0.572 | 0.973 |
| 12 | I am satisfied with my family life | | 3.90 | 0.347 | 3.81±0.472 | | 3.95±0.225 | 0.001α |
| ****JSF Average (total score) | | | 3.10 (37.2) | 0.591  (7.141) | 3.09±0.55 | | 3.10±0.58 | 0.072 |

WE: Work engagement, WH: Workaholism, JS: Job Stress, JSF: Job satisfaction. * Highly engaged (WE) score, **Highly workaholic (WH) score, *** Moderately stressed (JS) score, **** very satisfied (JSF
